# ER targeting of non-imported mitochondrial carrier proteins is dependent on the GET pathway

**DOI:** 10.26508/lsa.202000918

**Published:** 2021-01-21

**Authors:** Tianyao Xiao, Viplendra PS Shakya, Adam L Hughes

**Affiliations:** Department of Biochemistry, University of Utah School of Medicine, Salt Lake City, UT, USA

## Abstract

Deficiencies in mitochondrial import cause the toxic accumulation of non-imported mitochondrial precursor proteins. Numerous fates for non-imported mitochondrial precursors have been identified in budding yeast, including proteasomal destruction, deposition into protein aggregates, and mistargeting to other organelles. Amongst organelles, the ER has emerged as a key destination for a subset of non-imported mitochondrial proteins. However, how ER targeting of various types of mitochondrial proteins is achieved remains incompletely understood. Here, we show that the ER delivery of endogenous mitochondrial transmembrane proteins, especially those belonging to the SLC25A mitochondrial carrier family, is dependent on the guided entry of tail-anchored proteins (GET) complex. Without a functional GET pathway, non-imported mitochondrial proteins destined for the ER are alternatively sequestered into Hsp42-dependent protein foci. Loss of the GET pathway is detrimental to yeast cells experiencing mitochondrial import failure and prevents re-import of mitochondrial proteins from the ER via the ER-SURF pathway. Overall, this study outlines an important role for the GET complex in ER targeting of non-imported mitochondrial carrier proteins.

## Abstract (precis)

The GET pathway is required to target non-imported mitochondrial carrier proteins to the endoplasmic reticulum, which prevents their deposition into Hsp42-dependent protein foci.

## Introduction

Mitochondria play crucial roles in ATP production, metabolite synthesis, cell immunity, and apoptosis (Friedman & Nunnari, 2014). Abnormal mitochondrial function disrupts cellular homeostasis and is tightly linked to aging and many metabolic diseases (Wallace, 2005). A major consequence of mitochondrial dysfunction is the impairment of mitochondrial protein import. The vast majority of the mitochondrial proteome, which contains more than 1,000 proteins, is encoded in the nucleus and synthesized in the cytoplasm (Pagliarini etal, 2008). Mitochondrial precursor proteins are imported into mitochondria by translocase complexes located in the outer and inner mitochondrial membranes (OMM and IMM) (Wiedemann & Pfanner, 2017). The translocation of mitochondrial proteins containing mitochondrial targeting sequences is dependent on IMM potential (Wiedemann & Pfanner, 2017). Thus, in response to mitochondrial dysfunction, mitochondrial protein import is impaired and non-imported proteins accumulate outside of mitochondria (Hughes & Gottschling, 2012; Wang & Chen, 2015; Wrobel et al, 2015; Boos et al, 2020).

Previous studies found that non-imported mitochondrial proteins trigger proteotoxicity, initially termed mitochondrial precursor overaccumulation stress (Wang & Chen, 2015; Wrobel et al, 2015). To date, several studies have shown that mitochondrial protein-induced stress triggers a cascade of cellular responses that help to promote cellular survival, including translational suppression and proteasomal destruction in the cytoplasm, nucleus and at the mitochondrial surface (Wang & Chen, 2015; Wrobel et al, 2015; Itakura et al, 2016; Hansen et al, 2018; Mårtensson et al, 2019; Boos et al, 2020; Shakya et al, 2020 *Preprint*). In a recent screen to elucidate fates of non-imported mitochondrial proteins, we identified the ER as an organelle to which many non-imported mitochondrial membrane proteins were targeted (Shakya et al, 2020 *Preprint*). This observation is consistent with other studies that have also identified alternative targeting of mitochondrial proteins to the ER under a variety of conditions (Friedman et al, 2018; Hansen et al, 2018; Vitali et al, 2018; McKenna et al, 2020; Qin et al, 2020). Although these studies support the role of the ER as a major destination for non-imported mitochondrial proteins, our understanding of the mechanisms underlying alternative ER delivery of mitochondrial proteins remains incompletely understood. It was recently shown that the guided entry of tail-anchored proteins (GET) pathway, a known posttranslational ER insertion pathway for C-terminal tail-anchored (TA) proteins (Schuldiner et al, 2008), increases the risk of mistargeting of mitochondrial outer membrane proteins to the ER (Vitali et al, 2018). Interestingly, our prior screen identified a variety of different types of mitochondrial proteins that localized to the ER upon mitochondrial import failure outside of those identified as GET-dependent, including single-pass OMM proteins and single- and multi-pass IMM proteins (Shakya et al, 2020 *Preprint*). Thus, it remains an open question as to how ER targeting of all these various types of non-imported mitochondrial proteins is achieved.

Here, we sought to identify factors required for ER targeting of non-imported mitochondrial proteins during conditions of mitochondrial impairment. In synergy with previous observations (Vitali et al, 2018), we found that the GET complex is indispensable for targeting endogenous non-imported mitochondrial carrier proteins to the ER. Specifically, we find that Get3, the cytosolic ATPase of the GET pathway (Schuldiner et al, 2008), colocalizes with non-imported mitochondrial carrier proteins. In the absence of a functional GET pathway, ER-destined non-imported mitochondrial proteins instead localize to Hsp42-dependent cytosolic foci that associate with both mitochondria and the ER. We further show that in cells lacking core components of the GET pathway, pharmaceutical or genetic inhibition of mitochondrial protein import causes dramatically reduced cellular survival. In addition, GET-dependent ER-localized non-imported mitochondrial proteins are potential substrates for the ER-SURF pathway (Hansen et al, 2018) that promotes re-import of these proteins to mitochondria. Thus, it appears that the GET pathway plays a significant role in quality control of non-imported mitochondrial carrier proteins.

## Results

### A subset of non-imported mitochondrial proteins are targeted to the ER

**Figure 1.**
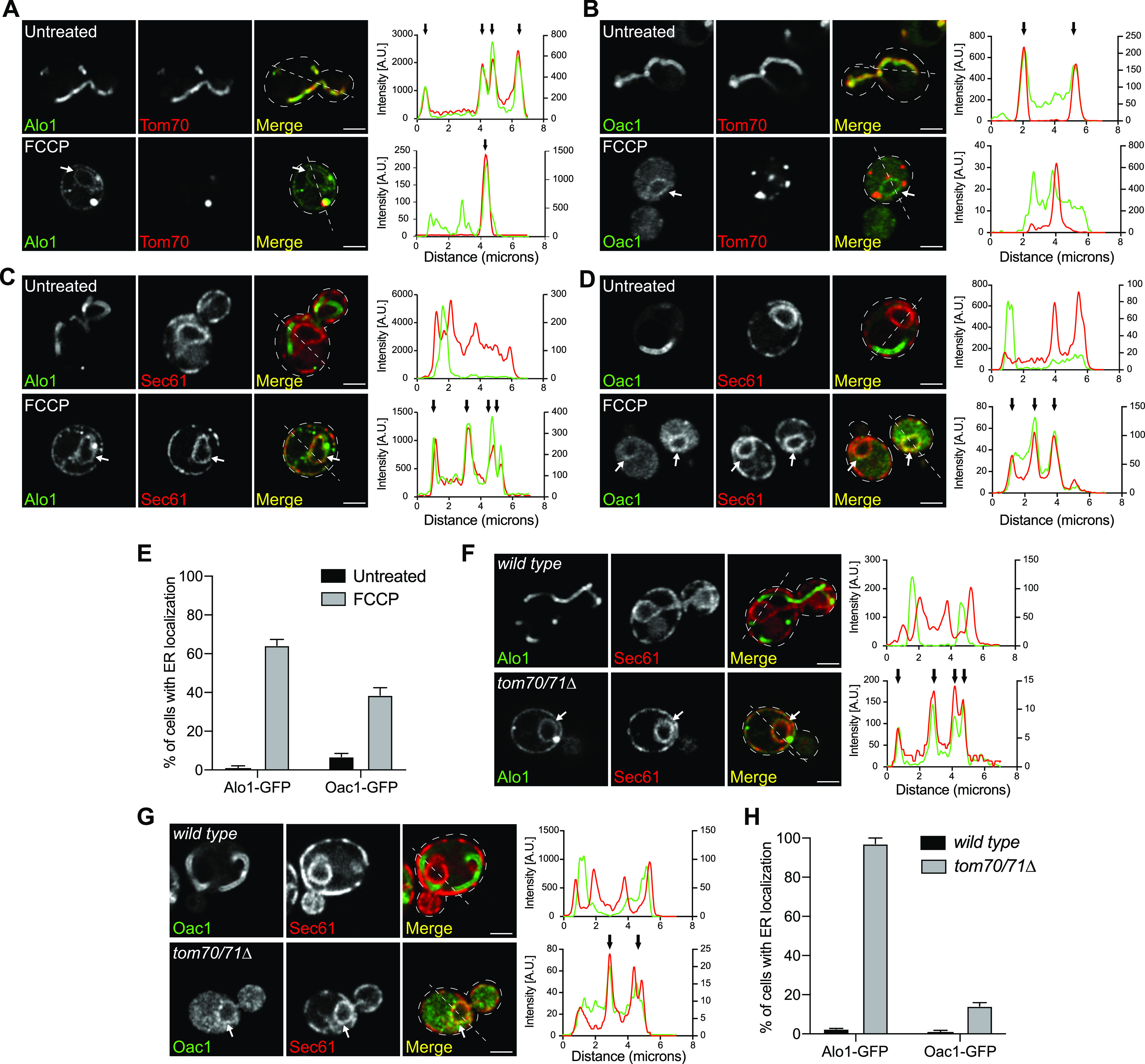
Non-imported mitochondrial proteins are targeted to the ER. **(A, B)** Super-resolution images and line scan analysis of yeast expressing Alo1-GFP (A) or Oac1-GFP (B) and Tom70-mCherry −/+ FCCP. **(C, D)** Super-resolution images and line scan analysis of yeast expressing Alo1-GFP (C) or Oac1-GFP (D) and Sec61-mCherry −/+ FCCP. **(E)** Quantification of cells with ER localization of Alo1- or Oac1-GFP −/+ FCCP. N > 100 cells per replicate, error bars = SEM of three replicates. **(F, G)** Super-resolution images and line scan analysis of wild type or *tom70/71*Δ expressing Alo1-GFP (F) or Oac1-GFP (G) and Sec61-mCherry. **(H)** Quantification of cells with ER localization of Alo1- or Oac1- GFP in wild-type cells or *tom70/71*Δ mutants. N > 100 cells per replicate, error bars = SEM of three replicates. For (A, B, C, D, F, G), white arrow marks perinuclear ER. White line marks fluorescence intensity profile position. Left and right y-axis (line scan graph) corresponds to GFP and mCherry fluorescence intensity, respectively. Black arrow (line scan graph) marks colocalization. Images show single focal plane. Scale bar = 2 μm. See also Figs S1 and S2.

**Figure S1.**
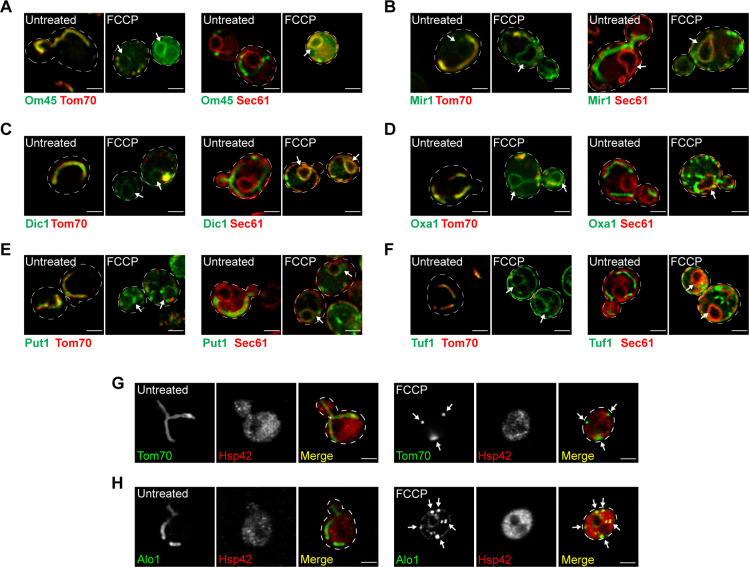
Additional non-imported mitochondrial proteins are targeted to the ER (related to Fig 1). **(A, B, C, D, E, F)** Super-resolution images of yeast expressing indicated outer mitochondrial membrane proteins (A), IMM proteins (B, C, D), or mitochondrial proteins (E, F) tagged with GFP and Tom70-mCherry/Sec61-mCherry −/+ FCCP. Sub-organelle localizations of mitochondrial proteins were obtained from SGD. White arrows mark perinuclear ER. **(G, H)** Super-resolution images of yeast expressing Tom70 (G) or Alo1 (H) tagged with GFP and Hsp42-mCherry −/+ FCCP. White arrows mark collapsed mitochondria upon FCCP treatment. All images show single focal plane. Scale bar = 2 μm.

**Figure S2.**
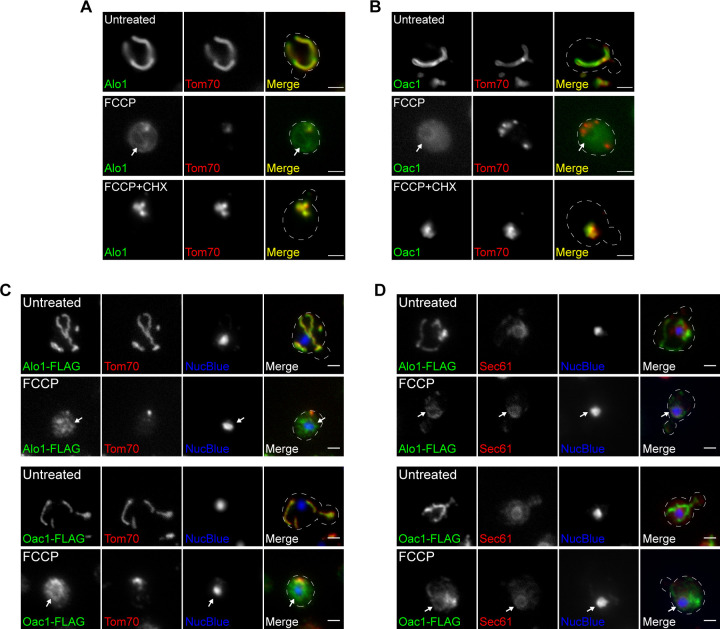
Newly synthesized non-imported mitochondrial proteins are targeted to the ER (related to Fig 1). **(A, B)** Wide-field images of yeast expressing Alo1-GFP (A) or Oac1-GFP (B) and Tom70-mCherry −/+ FCCP −/+ CHX (cycloheximide). White arrow marks perinuclear ER. **(C, D)** Wide-field images of indirect immunofluorescence staining against the FLAG epitope in yeast expressing Alo1- or Oac1-FLAG and Tom70-mCherry (C) or Sec61-mCherry (D) −/+ FCCP. Nucleus stained with NucBlue. White arrow marks perinuclear ER. Images show single focal plane. Scale bar = 2 μm.

We previously conducted a microscopy-based screen using the budding yeast GFP clone collection to study the localization and abundance of more than 400 mitochondrial proteins under conditions of mitochondrial membrane depolarization induced by the ionophore trifluoromethoxy carbonyl cyanide phenylhydrazone (FCCP) (Shakya et al, 2020 *Preprint*). Through the screen, the ER was identified as a destination for ∼3% of the non-imported mitochondrial proteome, in agreement with prior observations of mitochondrial proteins aberrantly localizing to the ER (Hansen et al, 2018; Vitali et al, 2018; McKenna et al, 2020; Qin et al, 2020). We verified the localization of eight ER-localized candidates using newly generated yeast strains in which mitochondrial proteins of interest were endogenously tagged with GFP at their C-termini, and the OMM protein Tom70 was fused to mCherry to mark mitochondria (Hughes & Gottschling, 2012; Hughes et al, 2016). Using super-resolution microscopy, we found that in untreated cells, all eight proteins localized to mitochondria as expected (Figs 1A and B and S1A–F). Upon FCCP treatment, the eight proteins of interest now localized to structures characteristic of yeast ER, in addition to residual localization to collapsed mitochondria fragments that we confirmed were not protein aggregates (Figs 1A and B and S1A–H) (Lee et al, 2018). Most of these proteins were mitochondrial membrane proteins, including both OMM proteins, for example, Alo1 (Fig 1A), and IMM proteins, for example, Oac1 (Fig 1B). ER localization of these mitochondrial proteins was confirmed by their colocalization with mCherry-tagged Sec61, a component of the ER-localized translocon (Young et al, 2012; Aviram & Schuldiner, 2017) (Figs 1C–E and S1A–F). In the presence of cycloheximide, which inhibits protein synthesis, ER localization of Alo1 and Oac1 was undetectable upon FCCP treatment (Fig S2A and B), indicating only newly synthesized Alo1 and Oac1 were targeted to the ER. C-terminal FLAG-tagged Alo1 and Oac1 were also targeted to the ER upon FCCP treatment as determined by indirect immunofluorescence, similar with their GFP-tagged counterparts (Fig S2C and D). Thus, ER localization of these mitochondrial proteins was not due to their C-terminal GFP fusion. In addition to FCCP, we also used genetic tools to specifically block mitochondrial import via deletion of *TOM70* and *TOM71*. Tom70 and Tom71 reside on the OMM and facilitate the import of both Alo1 and Oac1 (Wiedemann & Pfanner, 2017). In *tom70/tom71*Δ mutants but not in wild-type cells, Alo1-GFP and Oac1-GFP colocalized with Sec61-mCherry (Fig 1F–H). Although it remains unclear at this point whether these proteins are inserted to the ER or peripherally associated with the ER membrane, our data do confirm that several mitochondrial proteins are alternatively localized to the ER in response to either acute or constitutive mitochondrial import blockade.

### The GET complex is required for localization of non-imported mitochondrial carrier proteins to the ER

**Figure S3.**
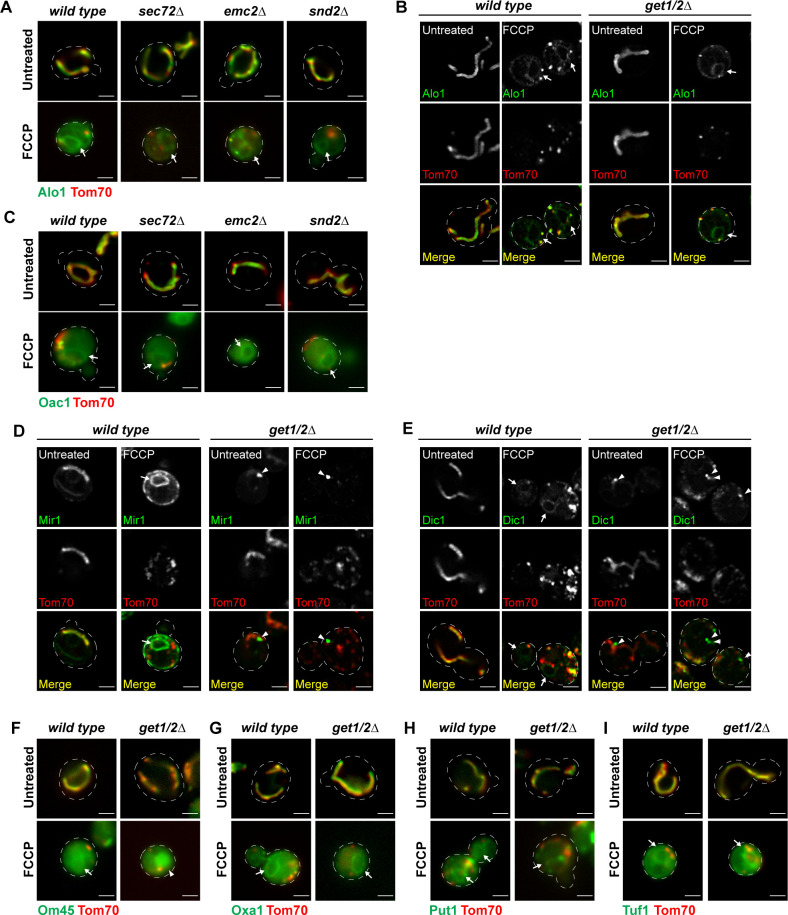
The GET complex selectively facilitates ER targeting of several non-imported mitochondrial proteins (related to Fig 2). **(A)** Wide-field images of wild-type or the indicated mutant yeast expressing Alo1-GFP and Tom70-mCherry −/+ FCCP. White arrow marks perinuclear ER. **(B)** Super-resolution images of wild-type or *get1/2*Δ mutant yeast expressing Alo1-GFP and Tom70-mCherry −/+ FCCP. White arrow marks perinuclear ER. **(C)** Wide-field images of wild-type or the indicated mutant yeast expressing Oac1-GFP and Tom70-mCherry −/+ FCCP. White arrow marks perinuclear ER. **(D, E)** Super-resolution images of wild-type or *get1/2*Δ mutant yeast expressing IMM carrier proteins Mir1-GFP (D) or Dic1-GFP (E) and Tom70-mCherry −/+ FCCP. White arrow marks perinuclear ER. White arrowhead marks protein foci containing GFP-tagged mitochondrial proteins. **(F)** Wide-field images of wild-type or *get1/2*Δ yeast expressing outer mitochondrial membrane protein Om45-GFP and Tom70-mCherry −/+ FCCP. White arrow marks perinuclear ER. White arrowhead marks vacuole. **(G, H, I)** Wide-field images of wild-type or *get1/2*Δ mutant yeast expressing IMM protein Oxa1-GFP (G) or mitochondrial protein Put1-GFP (H) or Tuf1-GFP (I) and Tom70-mCherry −/+ FCCP. White arrow marks perinuclear ER. Images show single focal plane. Scale bar = 2 μm.

**Figure 2.**
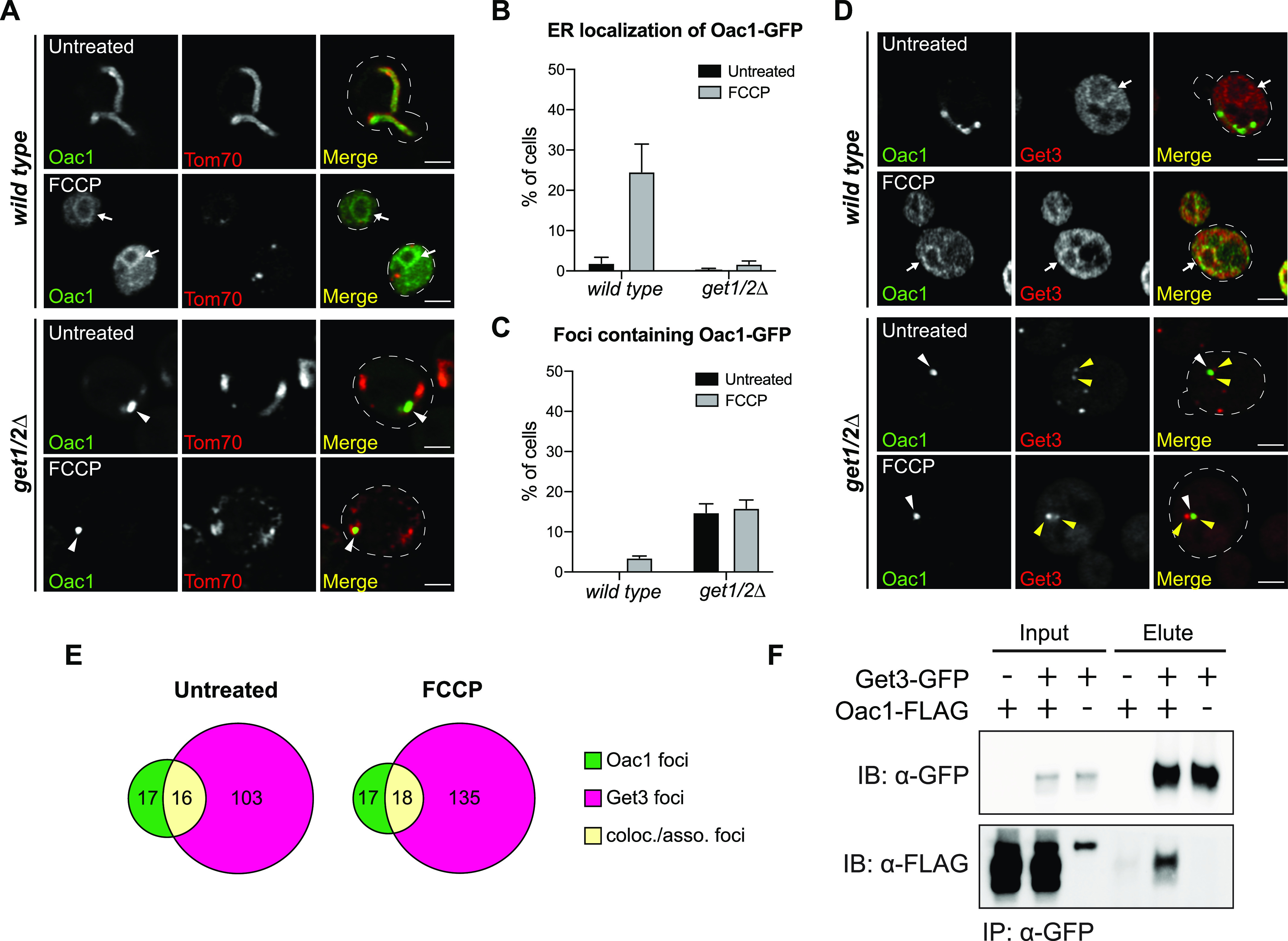
The GET complex is required for ER targeting of non-imported mitochondrial carrier proteins. **(A)** Super-resolution images of wild-type or *get1/2*Δ mutant cells expressing Oac1-GFP and Tom70-mCherry −/+ FCCP. White arrow marks perinuclear ER. White arrowhead marks protein foci containing Oac1-GFP. Images show single focal plane. **(B, C)** Quantification of (A) showing the percentage of cells with Oac1-GFP localized to the ER (B) or protein foci (C). N > 100 cells per replicate, error bars = SEM of three replicates. **(D)** Super-resolution images of wild-type or *get1/2*Δ cells expressing Oac1-GFP and Get3-mCherry −/+ FCCP. White arrow marks perinuclear ER. White arrowhead marks protein foci containing Oac1-GFP. Yellow arrowheads mark protein foci containing Get3-mCherry. Images show single focal plane. **(E)** Quantification of (D) showing the number of foci only containing Oac1-GFP (green), Get3-mCherry (magenta), or colocalized/associated Oac1-GFP and Get3-mCherry (yellow) per 100 cells −/+ FCCP. N > 100 cells per replicate of three replicates, values are normalized to number of foci per 100 cells. **(F)** Western blot probing for GFP and FLAG in input or elution products of immunoprecipitated Get3-GFP in the indicated yeast strains. Scale bar = 2 μm. See also Figs S3–S5.

To investigate the cellular machinery required for targeting these non-imported mitochondrial proteins to the ER, we surveyed non-imported mitochondrial protein localization by microscopy in a set of strains with deficiencies in known ER-import pathways, including the Sec61 translocon that imports ER proteins through either the signal recognition particle (SRP)-dependent or SRP-independent pathways, the ER membrane protein complex (EMC), the SRP-independent targeting (SND) complex, and the GET complex (Schuldiner et al, 2008; Ast & Schuldiner, 2013; Aviram et al, 2016; Aviram & Schuldiner, 2017; Chitwood et al, 2018; Guna et al, 2018; Shurtleff et al, 2018). Alo1 or Oac1 were endogenously tagged with GFP in mutants with deletion of either SRP-independent Sec61 translocon component *SEC72*, EMC component *EMC2*, SND complex components *SND2*, or GET pathway insertases *GET1/2* (Schuldiner et al, 2008; Wang et al, 2014a; Aviram et al, 2016; Shurtleff et al, 2018). In response to FCCP, the ER localization of Alo1 was unaffected in any of these mutants (Fig S3A and B). Likewise, the ER localization of Oac1 in *sec72*Δ, *emc2*Δ, and *snd2*Δ upon FCCP treatment was similar to wild type (Fig S3C). In contrast, an obvious reduction in FCCP-induced ER targeting of Oac1 was observed upon disruption of the GET pathway (Fig 2A and B), which normally facilitates post-translational insertion of TA proteins to the ER (Schuldiner et al, 2008). Interestingly, in *get1/2*Δ mutant cells, Oac1 was sequestered in bright protein foci that were distinct from Tom70-labeled mitochondria fragments (Fig 2A and C), consistent with previous observations that TA proteins localize to protein foci in GET mutants (Schuldiner et al, 2008; Powis et al, 2013). We examined additional ER-targeted non-imported mitochondrial proteins in cells lacking Get1/2 and found that the FCCP-induced ER targeting of Mir1 and Dic1, both members of the multi-pass mitochondrial carrier protein family like Oac1 (Palmieri et al, 2006), was also dependent on the GET pathway (Fig S3D and E). Om45, an OMM protein, localized to the vacuole instead of the ER in *get1/2*Δ mutants upon FCCP treatment (Fig S3F). In contrast, other ER-destined non-imported mitochondrial proteins still localized to the ER in GET-deficient cells when treated with FCCP (Fig S3G–I), suggesting that like Alo1, their targeting is independent of the GET machinery and that multiple mechanisms exist to target different non-imported proteins to the ER.

**Figure S4.**
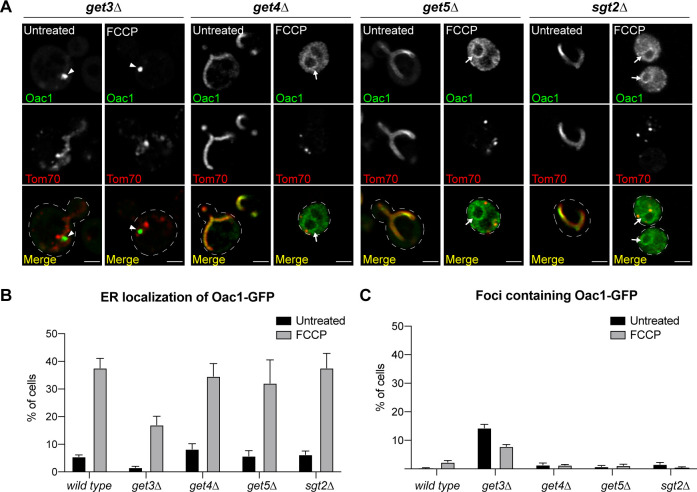
Get3 is partially required for ER delivery of Oac1-GFP (related to Fig 2). **(A)** Super-resolution images of the indicated mutant yeast expressing Oac1-GFP and Tom70-mCherry −/+ FCCP. White arrows mark perinuclear ER. White arrowhead marks protein foci containing Oac1-GFP. Images show single focal plane. Scale bar = 2 μm. **(B, C)** Quantification of (A) showing the percentage of cells with Oac1-GFP localized to the ER (B) or cytosolic foci (C) in wild-type or the indicated mutant cells. N > 100 cells per replicate, error bars = SEM of five replicates.

To further investigate the involvement of the GET complex in ER targeting of non-imported mitochondrial proteins, we tested the requirement of upstream GET components in delivery of Oac1 to the ER, including the cytosolic ATPase Get3, which binds and recruits substrates to the ER insertases Get1/2, and components of the pre-targeting complex Get4, Get5, and Sgt2, which bind and stabilize substrates to promote downstream ER targeting by Get1/2/3 (Wang et al, 2010, 2014a). Like Get1/2, loss of Get3 also impacted targeting of Oac1 to the ER (Fig S4A), with reduced ER localization upon FCCP treatment (Fig S4B) and appearance of non-mitochondrial Oac1 foci (Fig S4A and C). Knockout of *GET4*, *GET5*, or *SGT2*, however, had no effect on Oac1 localization (Fig S4A–C). This latter result is in line with previous studies showing that deletion of upstream factors of the GET complex, including Get4, Get5 or Sgt2, does not completely prevent functionality of the GET pathway (Kohl et al, 2011). Thus, core GET components, including Get1/2 and partially Get3, are required for targeting mitochondrial carrier proteins to the ER, but other components of the GET pathway are dispensable.

**Figure S5.**
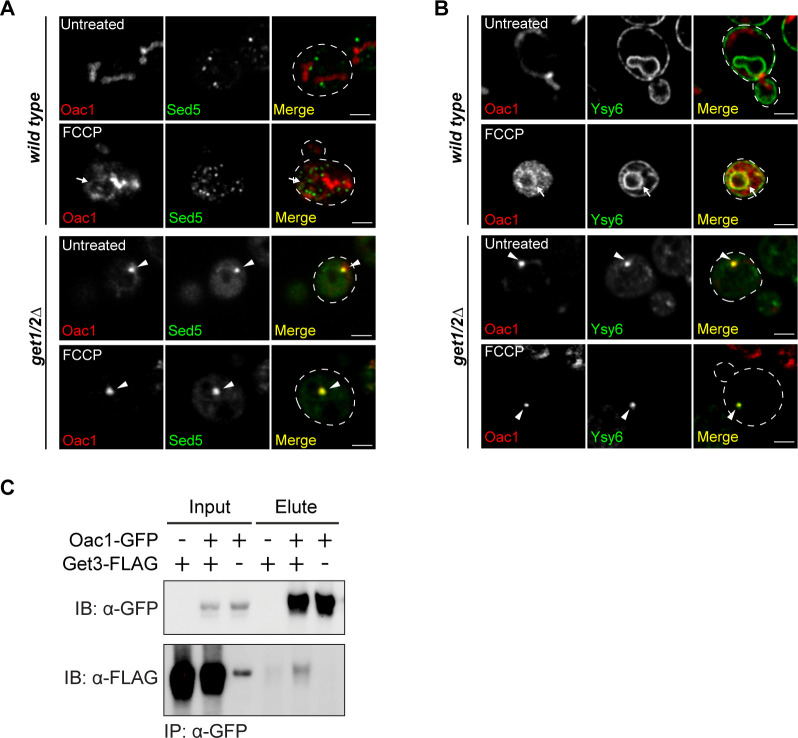
Oac1 colocalizes with Get3 and tail-anchored proteins in *get1/2*Δ mutant cells (related to Fig 2). **(A, B)** Super-resolution images of wild-type or *get1/2*Δ cells expressing Oac1-mCherry and GFP-Sed5 (A) or GFP-Ysy6 (B) −/+ FCCP. White arrow marks perinuclear ER. White arrowhead marks protein foci containing Oac1-mCherry. Images show single focal plane. Scale bar = 2 μm. **(C)** Western blot probing for GFP and FLAG in input or elution products of immunoprecipitated Oac1-GFP in the indicated yeast strains.

We also analyzed whether non-imported Oac1 colocalize with components of the GET machinery in cells. To do this, we created a strain expressing an mCherry-tagged version of Get3, the cytosolic ATPase that normally resides in the cytoplasm and recruits cytosolic GET substrates to ER-localized Get1/2 (Schuldiner et al, 2008; Wang et al, 2010). In *get1/2*Δ mutants, it has been shown that Get3 localizes to cytosolic foci containing GET substrates (Schuldiner et al, 2008; Powis et al, 2013). Like canonical TA substrates of the GET pathway (Powis et al, 2013), we found that in *get1/2*Δ mutant cells, half of the Oac1-GFP foci were colocalized or closely associated with Get3-mCherry foci, even in times where foci were observed in the absence of FCCP (Fig 2D and E). These foci also contained the TA proteins Sed5 and Ysy6 (Fig S5A and B), consistent with the idea that these puncta are the same as reported previously in *get1/2*Δ mutants (Schuldiner et al, 2008). Furthermore, co-immunoprecipitation analysis indicated that a small fraction of GFP-tagged Get3 constitutively co-purified with FLAG-tagged Oac1 (Fig 2F), which persisted regardless of the nature of the epitope tags on the protein, or which of the proteins was used as the bait (Fig S5C). Together, these data indicate that Oac1 associates with Get3 in cells, further supporting an interplay between the GET pathway and non-imported mitochondrial carrier proteins.

### Oac1 localizes to mitochondria- and ER-associated Hsp42-dependent foci in the absence of a functional GET pathway

**Figure 3.**
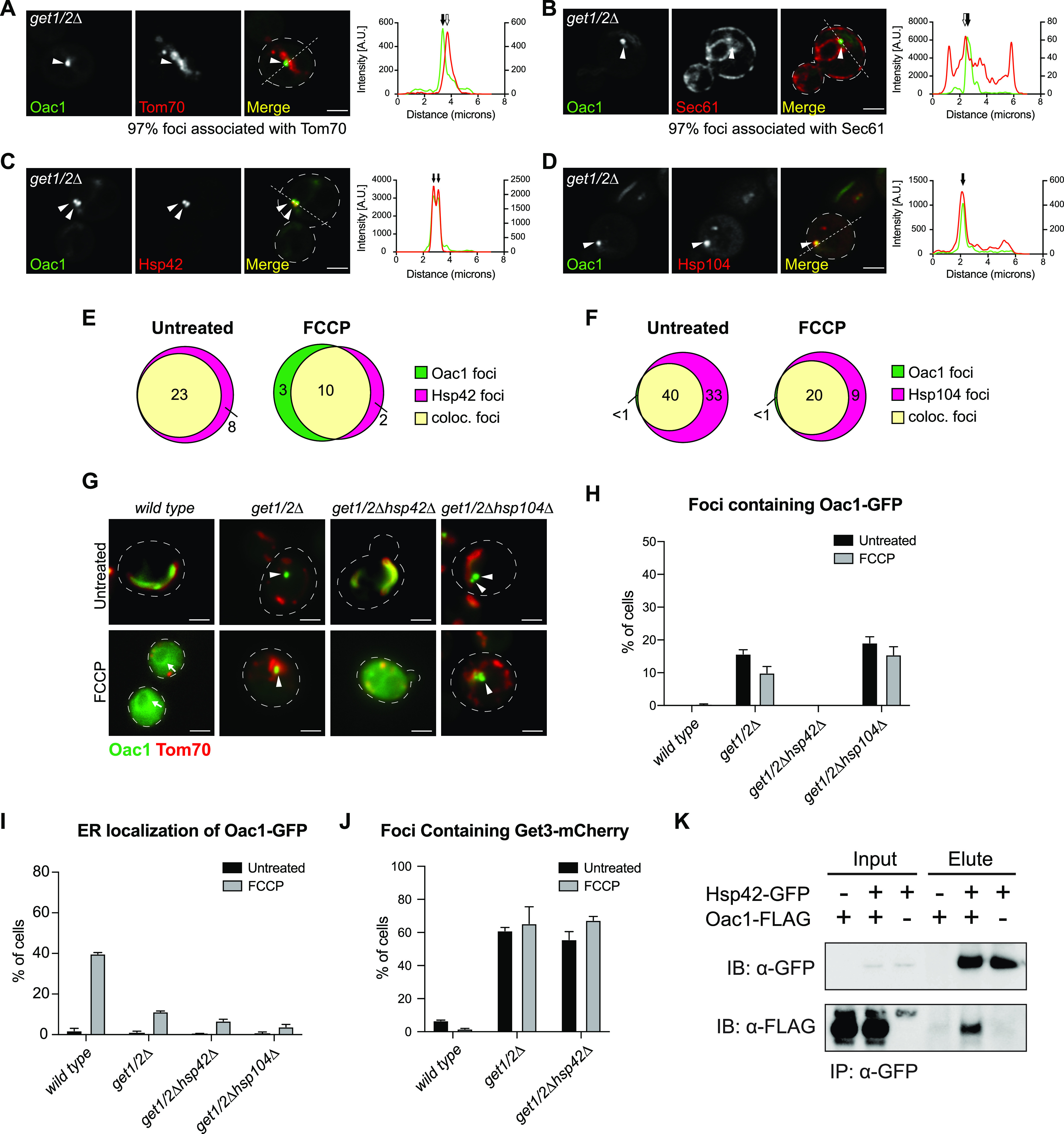
Oac1-GFP localizes to mitochondrion- and ER-associated Hsp42-dependent foci in the absence of a functional GET pathway. **(A, B, C, D)** Super-resolution images and line scan analysis of *get1/2*Δ mutant yeast expressing Oac1-GFP and Tom70-mCherry (A), Sec61-mCherry (B), Hsp42-mCherry (C), or Hsp104-mCherry (D). White arrowhead marks protein foci containing Oac1-GFP. For microscopy images, white line marks fluorescence intensity profile position. Images show single focal plane. Scale bar = 2 μm. For line scan graphs, Left and right y-axis correspond to GFP and mCherry fluorescence intensity, respectively. Black arrow marks protein foci position and white arrow marks mitochondria (A) or ER (B) position that is associated with protein foci. For the quantification in (A) and (B), N > 100 cells per replicate of three replicates. **(E, F)** Quantification of (C) and (D), respectively, showing the number of foci only containing Oac1-GFP (green), Hsp42-mCherry (E) or Hsp104-mCherry (F) (magenta) or both (yellow) per 100 cells −/+ FCCP. N > 100 cells per replicate of three replicates, values are normalized to number of foci per 100 cells. **(G)** Wide-field images of wild-type cells and the indicated mutant yeast expressing Oac1-GFP and Tom70-mCherry −/+ FCCP. White arrows mark perinuclear ER. White arrowheads mark protein foci containing Oac1-GFP. Images show single focal plane. Scale bar = 2 μm. **(H, I)** Quantification of (G) showing the percentage of cells with Oac1-GFP localized to protein foci (H) or the ER (I). N > 100 cells per replicate, error bars = SEM of three replicates. **(J)** Quantification of the percentage of cells with protein foci containing Get3-mCherry in wild-type or the indicated mutant cells. N > 100 cells per replicate, error bars = SEM of three replicates. **(K)** Western blot probing for GFP and FLAG in input or elution products of immunoprecipitated Hsp42-GFP in the indicated yeast strains.

In cells with a non-functional GET pathway, mitochondrial carrier proteins were sequestered into protein foci (Figs 2A and C, S3D and E, and S4A). We sought to further characterize the nature of these foci. To do this, we analyzed their localization in cells with fluorescently tagged organelle markers using super-resolution microscopy. In *get1/2*Δ mutant cells, 97% of protein foci containing Oac1 were associated with mitochondria marked by Tom70 (Fig 3A) or the ER marked by Sec61 (Fig 3B), which is similar with previously characterized cytosolic protein aggregates (Zhou et al, 2014). To verify whether these foci corresponded to protein aggregates, we labeled Hsp42 and Hsp104, chaperones that commonly localize to cytosolic protein aggregates in yeast (Zhou et al, 2014; Miller et al, 2015; Lee et al, 2018), with mCherry and examined localization with Oac1-GFP foci. We found that nearly all Oac1-GFP foci contained Hsp42 and Hsp104, even in untreated cells (Fig 3C–F). Deletion of *HSP42*, but not *HSP104*, diminished the formation of Oac1 foci in *get1/2*Δ mutants (Fig 3G and H), leading to predominantly diffuse cytoplasmic localization (Fig 3G and I). Interestingly, the formation of Get3-foci in *get1/2*Δ was unaffected by *HSP42* deletion (Fig 3J), indicating that Hsp42 is required for Oac1 deposition into Get3-containing protein foci. Consistent with an important role for Hsp42 in the handling of non-imported Oac1, co-immunoprecipitation analysis identified an interaction between that FLAG-tagged Oac1 and GFP-tagged Hsp42 (Fig 3K). Thus, Hsp42 mediates sequestration of non-imported Oac1 into protein foci in the absence of a functional GET pathway.

### Dual loss of the GET pathway and mitochondrial import is detrimental to cells

**Figure 4.**
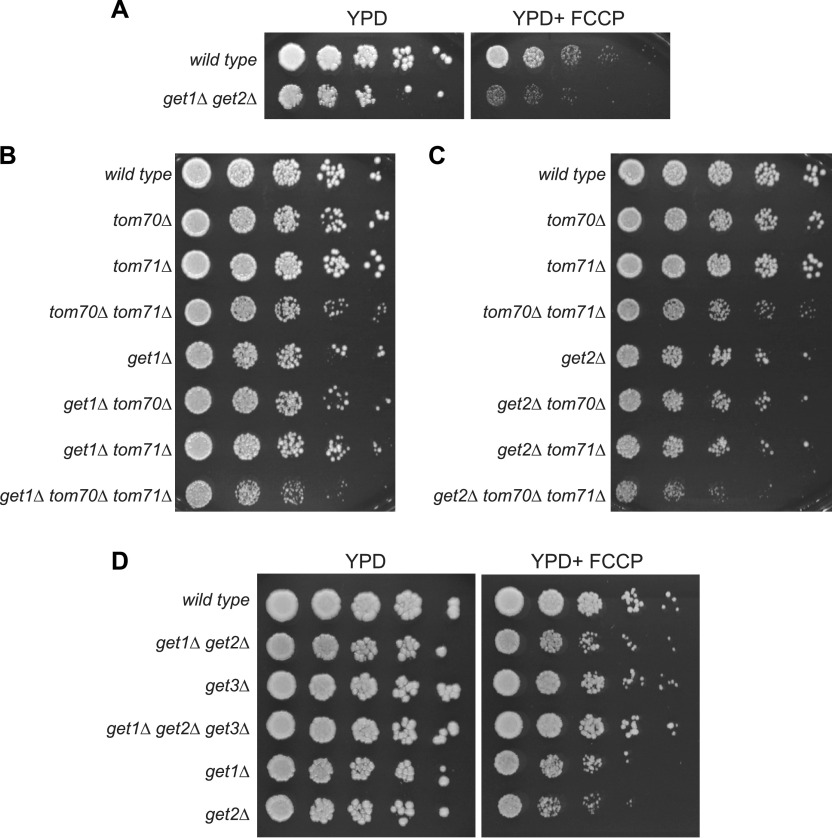
Deletion of *GET1/2* impairs growth of yeast cells with mitochondrial import failure. **(A)** Fivefold serial dilutions of wild-type cells and *get1/2*Δ mutant cells on YPD −/+ FCCP agar plates. **(B, C)** Fivefold serial dilutions of wild-type and the indicated mutant cells on YPD agar plates. **(D)** Fivefold serial dilutions of wild-type cells and the indicated mutant cells on YPD −/+ FCCP agar plates.

Because non-imported mitochondrial proteins are harmful to cells (Wang & Chen, 2015; Wrobel et al, 2015; Boos et al, 2020), we investigated whether loss of ER targeting of non-imported mitochondrial proteins during times of mitochondrial deficiency led to reduced cellular fitness. To do this, we tested the growth of GET mutants under stress of mitochondrial import failure. In comparison to wild-type cells, *get1/2*Δ cells exhibited more severely diminished growth in the presence of FCCP (Fig 4A). Likewise, cells lacking the mitochondrial import receptors Tom70/71 showed stronger fitness defects when combined with deletion of *GET1* or *GET2* (Fig 4B and C). Interestingly, the growth deficiencies of *get1/2*Δ mutant cells in the presence of FCCP were largely suppressed by deletion of *GET3* (Fig 4D), suggesting the presence of Get3 in the cytoplasm without its ER receptors is problematic under these conditions. Overall, these results suggest that dual loss of mitochondrial import and GET-dependent ER targeting is problematic for cells. At this point, it remains unclear to what extent these growth defects result from failure to target non-imported proteins to the ER in GET mutants versus other possibilities explanations, including altered targeting of TA proteins to the mitochondria in GET mutants, loss of proteostasis due to impaired biogenesis of other stress-responsive factors, or the presence of TA proteins and Get3 in the cytosol (Schuldiner et al, 2008; Jonikas et al, 2009).

### GET-dependent ER-destined mitochondrial proteins are potential substrates for the ER-SURF pathway

**Figure 5.**
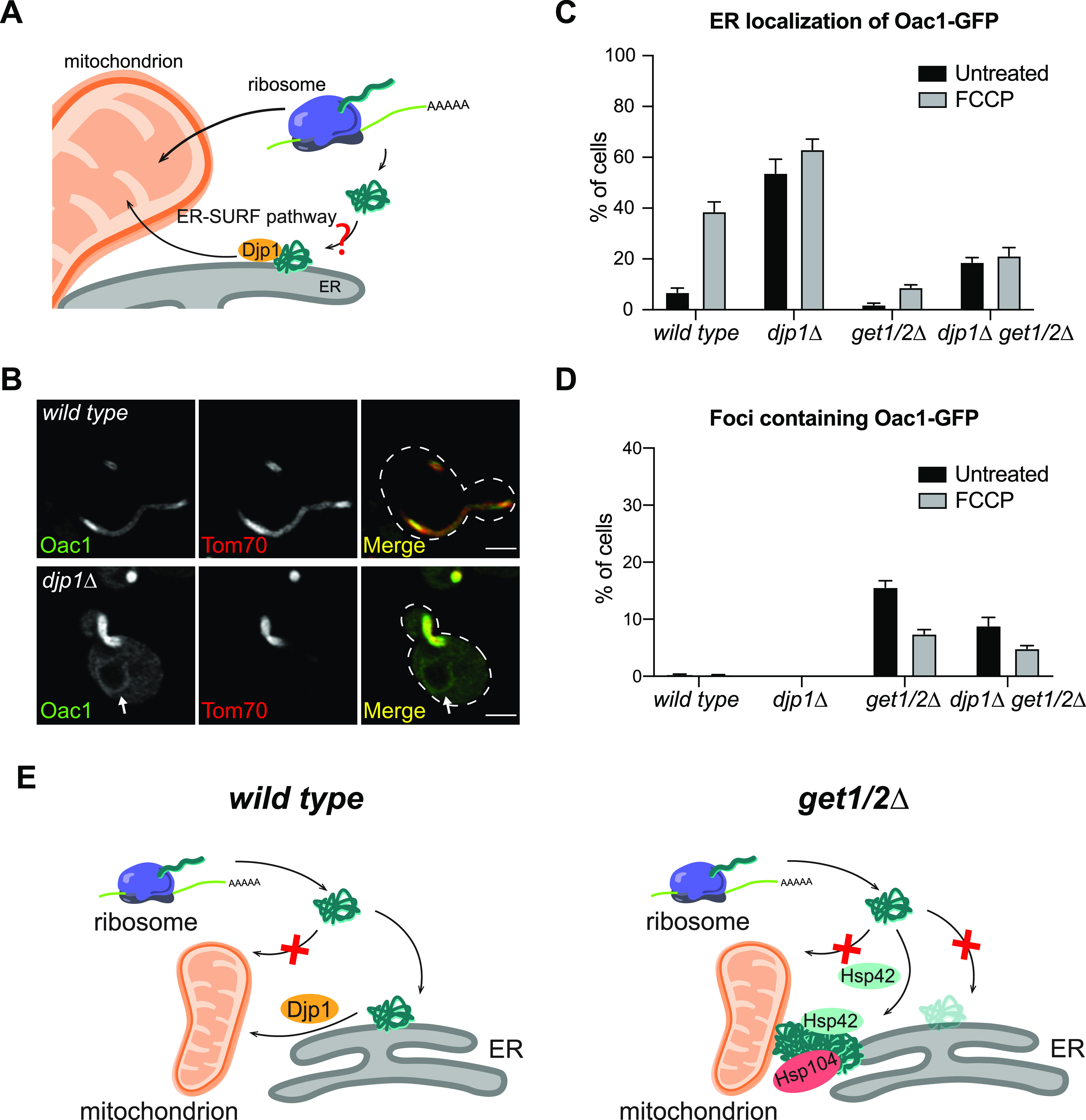
GET-dependent ER targeting of mitochondrial proteins potentially provides substrates for the ER-SURF pathway. **(A)** Schematic graph of the ER-SURF pathway. **(B)** Super-resolution images of wild-type and *djp1*Δ cells expressing Oac1-GFP and Tom70-mCherry. White arrows mark perinuclear ER. Images show single focal plane. Scale bar = 2 μm. **(C, D)** Quantification of the percentage of cells with Oac1-GFP localized to the ER (C) or the cytosolic foci (D) in wild-type or the indicated mutant cells. N > 100 cells per replicate, error bars = SEM of three replicates. **(E)** Schematic overview of the fates of non-imported mitochondrial carrier proteins in wild-type and *get1/2*Δ mutant cells.

In agreement with our current findings, it was recently demonstrated that a J-protein, Djp1, shuttles ER-localized mitochondrial proteins from the ER membrane to mitochondria, promoting additional attempts of mitochondrial import (Hansen et al, 2018). A question surrounding this pathway, termed ER-SURF, is the nature of the cellular machinery that promotes initial targeting of mitochondrial proteins to the ER (Fig 5A). With our discovery that ER targeting of non-imported mitochondrial membrane proteins is perturbed in GET mutants, we wondered whether the loss of the GET machinery limits accessibility of the ER-SURF pathway to non-imported mitochondrial precursor substrates. To test whether GET-dependent delivery of non-imported mitochondrial proteins to the ER is an upstream step for mitochondrial re-import mediated by Djp1, we tagged Oac1, which requires the GET complex to be localized to the ER, with GFP, in *djp1*Δ mutant cells. Interestingly, ER-localized Oac1 was observed in 55% of *djp1*Δ cells without FCCP treatment (Fig 5B and C) and more than 60% with FCCP treatment (Fig 5C). Both rates are higher than observed in wild-type cells (Fig 5C). This result, combined with the fact that Oac1 colocalizes with Get3 (Figs 2D–F and S5C) and localizes to Hsp42-dependent protein foci in *get1/2*Δ cells even without FCCP treatment (Fig 3), suggests that a portion of Oac1 is constitutively targeted to the ER and shuttled to the mitochondria through the ER-SURF pathway. Consistent with this hypothesis, the ER localization of Oac1 in *djp1*Δ cells was dramatically reduced in the absence of *GET1/2* (Fig 5C), and protein foci containing Oac1 were present in *djp1*Δ*get1/2*Δ triple mutants (Fig 5D). These data support a model in which non-imported mitochondrial proteins are delivered to the ER in a GET-dependent manner for mitochondrial re-import via the ER-SURF pathway.

## Discussion

We previously identified the ER as a destination for a subset of non-imported mitochondrial membrane proteins during times of mitochondrial dysfunction (Shakya et al, 2020 *Preprint*). Our current work now shows that the GET complex is required for ER targeting of a specific group of proteins, the mitochondrial carrier proteins. In cells with a dysfunctional GET pathway, the ER delivery of mitochondrial carrier proteins is impaired, and these proteins are instead sequestered into Hsp42-dependent mitochondrion- and ER-associated cytosolic protein foci (Fig 5E). Overall, our data support a requirement for the GET pathway in targeting of non-imported mitochondrial carrier proteins to the ER.

This work synergizes well with a recent report that showed that the GET complex increases the risk of mistargeting over-expressed OMM proteins to the ER (Vitali et al, 2018). Interestingly, similar to these previously described OMM proteins, multi-pass mitochondrial carrier proteins that require the GET pathway for ER delivery, including Oac1, Mir1, and Dic1, all contain transmembrane domains that are very close to their C-termini (Kunji, 2004). This might provide possibilities for their recognition by components of the GET complex. In comparison, Alo1, which likely contains a central transmembrane domain (Weill et al, 2019), is targeted to the ER independently of the GET pathway. However, it is important to emphasize that at this point, it remains unclear whether the GET pathway directly binds and facilitates targeting of multi-pass mitochondrial membrane proteins to the ER, or whether the role of the GET pathway in ER delivery of these proteins is indirect. While we find that Oac1 colocalizes with Get3 and TA proteins in cells lacking *GET1/2*, the nature of the interaction between Oac1 and Get3 is still obscure. Get3 is shown to have dual roles: as an ATPase that hands over clients to Get1/2 for further insertion (Schuldiner et al, 2008), and as a holdase chaperone that brings substrates to the ER and supports sequestration of substrates to protein foci in the absence of Get1/2 (Powis et al, 2013). Given that non-imported mitochondrial carrier proteins are potentially re-routed to mitochondria through the ER-SURF pathway, it is possible that Get3 acts as a holdase that facilitates ER targeting, but not translocation, of non-imported mitochondrial proteins. Thus, additional studies are required to determine whether non-imported mitochondrial proteins are inserted into the ER, how Get3 interacts with non-imported mitochondrial proteins, as well as whether Get1/2 play any direct role in importing Oac1 into the ER. In the absence of these experiments, it remains possible that the delivery of mitochondrial carrier proteins to the ER is mediated by an unknown pathway and that the block in ER delivery of these proteins in GET mutants may result from a general perturbation in cellular proteostasis (Jonikas et al, 2009). It is also possible that non-imported mitochondrial proteins might be targeted to the ER by unknown ER-localized factors that are originally inserted by the GET pathway. Deciphering between these possibilities will be an important avenue of research moving forward.

Despite the open questions surrounding the interplay between the GET pathway and non-imported mitochondrial proteins, our studies ultimately provide an important step forward in our understanding of how cells mitigate mitochondrial protein-induced stress. It is becoming clearer that cells use a multitude of pathways to prevent toxicity associated with the accumulation of non-imported mitochondrial proteins, and our work helps to further delineate the systems that promote delivery of mitochondrial membrane proteins to the ER. Interestingly, we also found that several ER-destined mitochondrial proteins did not appear to be affected by deletion of *GET1/2* for their ER targeting, suggesting that additional ER targeting systems for mitochondrial proteins likely exist. Identifying these systems and dissecting the coordination between the many cellular pathways that mitigate mitoprotein-induced stress will be critical areas of future research.

## Materials and Methods

### Reagents

Antibodies and chemicals used in this study are listed in Table S1.

Table S1 Antibodies, plasmids, and chemicals used in this study.

### Yeast strains

All yeast strains are derivatives of *Saccharomyces cerevisiae* S288c (BY) (Brachmann et al, 1998) and are listed in Table S2. Strains expressing tagged proteins from their native loci were created by one step PCR-mediated C-terminal endogenous epitope tagging using standard techniques and the oligo pairs listed in Table S3 (Brachmann et al, 1998; Sheff & Thorn, 2004). Plasmid templates for GFP tagging were from the pKT series of vectors (Sheff & Thorn, 2004). Plasmid templates for mCherry tagging were from the pKT series of vectors (Sheff & Thorn, 2004) or pFA6a-mCherry-HphMX (39295; Addgene) (Wang et al, 2014b). Integrations were confirmed by correct localized expression of the fluorophore by microscopy. Plasmid template for FLAG tagging was the pFA6a-5FLAG-KanMX6 (15983; Addgene) (Noguchi et al, 2008). Integrations were confirmed by a combination of colony PCR across the chromosomal insertion site and correct band size by Western blot. Deletion strains were created by one step PCR-mediated gene replacement using the oligos pairs listed in Table S3 and plasmid templates from the pRS series vectors (Brachmann et al, 1998). Correct integrations were confirmed with colony PCR across the chromosomal insertion site.

Table S2 Yeast strains used in this study.

Table S3 Oligos used in this study.

### Plasmids

Plasmids used in this study are listed in Table S1. Plasmids for GPD-driven expression of GFP-SED5 and GFP-YSY6 were generated by gateway-mediated transfer of the corresponding ORF (Harvard Institute of Proteomics) from pDONR201/221 into pAG413GPD-eGFP-ccdB (14310; Addgene), using Gateway LR Clonase II Enzyme mix (Thermo Fisher Scientific) according to the manufacturer’s instructions.

### Yeast cell culture and media

Yeast cells were grown exponentially for 15–16 h at 30°C to a final density of 2–7 × 10^6^ cells/ml before starting any treatments. Cells were cultured in YPAD medium (1% yeast extract, 2% peptone, 0.005% adenine, and 2% glucose) in most experiments. Cells with yeast plasmids expressing histidine auxotrophic marker genes were cultured in SD-His medium (0.67% yeast nitrogen base without amino acids, 2% glucose, supplemented nutrients 0.074 g/l each adenine, alanine, arginine, asparagine, aspartic acid, cysteine, glutamic acid, glutamine, glycine, myoinositol, isoleucine, lysine, methionine, phenylalanine, proline, serine, threonine, tryptophan, tyrosine, uracil, valine, 0.369 g/l leucine, and 0.007 g/l para-aminobenzoic acid). For FCCP or CHX (cycloheximide) treatment, overnight log-phase cell cultures were grown in the presence of FCCP (final concentration of 10 μM) or CHX (final concentration of 100 μg/ml) for 4–5 h.

### Microscopy

Optical z-sections of live yeast cells were acquired with a ZEISS Axio Imager M2 equipped with a ZEISS Axiocam 506 monochromatic camera, 100× oil-immersion objective (plan apochromat, NA 1.4), a AxioObserver 7 (Carl Zeiss) equipped with a PCO Edge 4.2LT Monochrome, Air Cooled, USB 3 CCD camera with a Solid-State Colibri 7 LED illuminator and 100× oil-immersion objective (Plan Apochromat, NA 1.4; Carl Zeiss), a ZEISS LSM800 equipped with an Airyscan detector, 63× oil-immersion objective (plan apochromat, NA 1.4) or a ZEISS LSM880 equipped with an Airyscan detector, 63× oil-immersion objective (plan apochromat, NA 1.4). Widefield images were acquired with ZEN (Carl Zeiss) and processed with Fiji (Schindelin et al, 2012). Super-resolution images were acquired with ZEN (Carl Zeiss) and processed using the automated Airyscan processing algorithm in ZEN (Carl Zeiss) and Fiji. Individual channels of all images were minimally adjusted in Fiji to match the fluorescence intensities between channels for better visualization. Line scan analysis was performed on non-adjusted, single z-sections in Fiji. All images shown in Figures represent a single optical section.

### Serial-dilution growth assays

Fivefold serial dilutions of exponentially growing yeast cells were diluted in ddH_2_O and 3 μl of each dilution was spotted onto YPD (1% yeast extract, 2% peptone, and 2% glucose). Final concentration of FCCP is 7 μM. Total cells plated in each dilution spot were 5,000, 1,000, 20, 40, and 8. Plates were cultured at 30°C for 36 h before obtaining images.

### Immunoprecipitation and Western blotting

Cells were grown as described above. 1 × 10^8^ total cells were harvested, resuspended in 500 μl of IP Buffer. Tris IP Buffer (50 mM Tris, pH 7.5, 150 mM NaCl, 1 mM EDTA, 10% Glycerol, 1% IGEPAL [NP-40 substitute], 100 μM PMSF, 1× cOmplete Protease Inhibitor Cocktail [Roche]) was used for immunoprecipitation with Hsp42, as previously described (Malinovska et al, 2012). KHM IP Buffer (110 mM KAc, 20 mM HEPES-KOH, pH 7.4, 2 mM MgCl_2_, 10% glycerol, 0.1% Triton-X100, 100 μM PMSF, and 1× cOmplete Protease Inhibitor Cocktail [Roche]) was used for immunoprecipitation of Oac1/Get3, adapted based on previous studies (Stefanovic & Hegde, 2007; Vitali et al, 2018). Resuspended cells were lysed with glass beads using an Omni Bead Ruptor 12 Homogenizer (eight cycles of 20 s each). Cells lysates were cleared by centrifugation at 10,000 rpm (Eppendorf 5424 Centrifuge) for 5 min to remove cell debris. The supernatant was collected in a new tube, and the total volume was adjusted to 1 ml by adding IP Buffer. Lysates were incubated with 2 μl of mouse anti-GFP antibodies (Sigma Millipore) at 4°C overnight. 50 μl of the lysate–antibody mixture was removed as input fraction. For each immunoprecipitation, 40 μl of Dynabeads Protein G (Thermo Fisher Scientific) were washed three times, and resuspended in 50 μl IP Buffer. Lysate–antibody mixture was incubated with washed Dynabeads Protein G at 4°C for 2 h and then washed four times for 10 min in IP Buffer. Immunoprecipitated proteins were eluted by incubating beads in 2× Laemmli Buffer (63 mM Tris, pH 6.8, 2% [wt/vol] SDS, 10% [vol/vol] glycerol, and 1 mg/ml bromophenol blue) at 65°C for 10 min.

Western blots were carried out as described previously (Hughes et al, 2016; Shakya et al, 2020 *Preprint*). Cells extracts and elution products were resolved on Bolt 4–12% Bis-Tris Plus Gels (NW04125BOX; Thermo Fisher Scientific) with NuPAGE MES SDS Running Buffer (NP0002-02; Thermo Fisher Scientific) and transferred to nitrocellulose membranes. Membranes were blocked and probed in blocking buffer (1× PBS, 0.05% Tween-20, 5% non-fat dry milk) using the primary antibodies for FLAG (Thermo Fisher Scientific) and GFP (Sigma Millipore) and HRP conjugated secondary antibodies (715-035-150; Jackson Immunoresearch). Blots were developed with SuperSignal West Dura Extended Duration Substrate (34075; Thermo Fisher Scientific) and exposed with a Bio-Rad Chemidoc MP system.

### Yeast indirect immunofluorescence (IIF) staining

For IIF staining, overnight log-phase cell cultures were grown with or without FCCP for 3 h 30 min in YPAD to a final density of 4 × 10^6^ cells/ml. Cells were harvested by centrifugation and fixed in 10 ml fixation medium (4% paraformaldehyde in YPAD) for 1 h. Fixed yeast cells were washed with Wash Buffer (0.1 M Tris, pH = 8, 1.2 M Sorbitol) twice and incubated in 2 ml DTT Buffer (10 mM DTT in 0.1 M Tris, pH = 9.4) at room temperature for 10 min. Spheroplasts were generated by incubating cells in 2 ml Zymolyase Buffer (0.1 M KPi, pH = 6.5, 1.2 M Sorbitol, 0.25 mg/ml Zymolyase) at 30°C for 30 min. Spheroplasts were gently diluted in 1:40 using Wash Buffer and attached to glass slides pre-coated with 0.1% poly-L-Lysine (2 mg/ml). Samples were permeabilized in cold 0.1% Triton X-100 in PBS for 10 min at 4°C, briefly dried, and blocked in Wash Buffer containing 1% BSA at room temperature for 30 min. After blocking, samples were incubated with primary antibody (Monoclonal ANTI-FLAG M2 antibody produced in mouse, 1:200 diluted in Wash Buffer containing 1% BSA) for 1 h 30 min at room temperature and secondary antibody (Goat anti-Mouse IgG (H+L) Cross-Adsorbed Secondary Antibody, Alexa Fluor 488, 1:300 diluted in Wash Buffer containing 1% BSA) for 45 min at room temperature. Samples were washed 10 times after each incubation with Wash Buffer containing 1% BSA and 0.1% Tween-20. Slides were washed twice with Wash Buffer before sealing, and mounted with HardSet medium (ProLong Glass Antifade Mountant with NucBlue Stain (P36981); Invitrogen) overnight. Wide-field images were acquired as described above.

### Quantification and statistical analysis

The number of replicates, what *n* represents, and dispersion and precision measures are indicated in the figure legends. In general, quantifications show the mean ± standard error from three biological replicates with *n* = 100 cells per experiment. In experiments with data depicted from a single biological replicate, the experiment was repeated with the same results.

## Supplementary Material

Reviewer comments
