# Supplementary material for: ER targeting of non-imported mitochondrial carrier proteins is dependent on the GET pathway
Source: Life Sci Alliance. 2021 Jan 21;4(3):e202000918. doi: 10.26508/lsa.202000918 (PMC7898604; doi:10.26508/lsa.202000918)
Supplement: Reviewer comments [file LSA-2020-00918_review_history.pdf]

Life Science Alliance

ER-targeting of non-imported mitochondrial carrier proteins is dependent on the GET pathway

Tianyao Xiao, Viplendra P.S. Shakya and Adam L Hughes
DOI: https://doi.org/10.26508/lsa.202000918

Corresponding author(s): Adam L Hughes, University of Utah

Review Timeline:

Submission Date:	2020-09-25
Editorial Decision:	2020-09-28
Revision Received:	2020-12-08
Editorial Decision:	2021-01-04
Revision Received:	2021-01-06
Accepted:	2021-01-06

Scientific Editor: Shachi Bhatt

Transaction Report:

(Note: With the exception of the correction of typographical or spelling errors that could be a source of ambiguity, letters and reports are not edited. The original formatting of letters and referee reports may not be reflected in this compilation.)

No full Peer Review Process File is available with this article, as the authors have chosen not to make the review process public in this case.

1st Editorial Decision September 28, 2020

Re: Life Science Alliance manuscript #LSA-2020-00918-T

Dr. Adam L Hughes
University of Utah Biochemistry
15 N Medical Drive East RM 4100
Salt Lake City, Utah 84112

Dear Dr. Hughes,

Thank you for transferring your manuscript entitled "The GET pathway safeguards against non-imported mitochondrial protein stress" to Life Science Alliance (LSA).

For a brief overview, the manuscript was reviewed at a partner journal, where the reviewers were not convinced that the imaging data alone were sufficient to support the conclusions. The authors transferred the manuscript and the reviewers comments to LSA. Given the intriguing data supporting the hypothesis that GET pathway is involved in ER targeting of non-imported mitochondrial proteins, the manuscript was considered appropriate for LSA after following edits:

+ Textual and Discussion requests:
++ The concerns raised by R1 and R2 (R1 pt 1, and R2 main point) about the use of microscopy data for determining the ER transport of non-imported mt proteins is valid, but does not need to be addressed experimentally. LSA would request you to tone down the conclusions pertaining to the mechanism claims; instead we request you to clarify the alternate hypotheses that could also explain the observed phenotypes
++ Please clarify R1's concerns about the diagram in Fig 4
++ The concerns raised by R2 under "Other specific concerns" should also be addressed in Discussion
++ Please edit line 139-140 as requested by R3 (R3 pt3)

+ Experimental requests:
++ Please address the technical issues pointed out by Rev 1 with regards to the Oac1-GET3 co-IP interaction (R1 pt 2)
++ R3 pt 1 (Fig 1), pt 2 (line 123-126), pt 7 (line 185-186), pt 8 (Fig 4A-B) and pt 9 (Fig 4H) should be addressed as they are directly related to the main message of the study

+ Good to have, but not required:
++ If possible, it would be great to show that deletion of other genes that have similarly strong proteostasis defects do not synergise with mitochondrial import failure (R1 pt 3). Please provide these data only if readily available, otherwise, we would appreciate a discussion of these concerns
++ If possible, we encourage you to provide data of Get5 rescue in get5Δ cells to show that ER localization of Oac1 is indeed restored in this strain (R3, pt 4, Fig S3A-C). If unable to provide this, we would appreciate a discussion of why the get4Δ, get5Δ show different results despite acting as a dimer; and tone down the conclusions accordingly
++ Please include the reference Kohl et al., 2011 and discuss their findings (supporting the paper's results) as suggested by R3 (R3, pt 5, line 148)
++ If possible, please provide data from get1/2/4 deletion showing a prevention of Get3 foci to support the

statements on physical interaction (R3 pt 6, line 160-163). If unable to include this data, please tone down the conclusions suggesting a physical interaction and only mention co=localization. - Addressing this point in one way or another would be required as the point was raised by both R1 and R3

We request you to submit a detailed point-by-point rebuttal along with the revised manuscript. We realize that such a revision would necessitate re-review. We would, of course, explain the transfer situation and the revision requirements to the reviewer / arbitrating reviewer in this case, if the transfer is sent to LSA.

To upload the revised version of your manuscript, please log in to your account:
https://lsa.msubmit.net/cgi-bin/main.plex
You will be guided to complete the submission of your revised manuscript and to fill in all necessary information. Please get in touch in case you do not know or remember your login name.

We would be happy to discuss the individual revision points further with you should this be helpful.

While you are revising your manuscript, please also attend to the below editorial points to help expedite the publication of your manuscript. Please direct any editorial questions to the journal office.

The typical timeframe for revisions is three months. Please note that papers are generally considered through only one revision cycle, so strong support from the referees on the revised version is needed for acceptance.

When submitting the revision, please include a letter addressing the reviewers' comments point by point.

Thank you for considering Life Science Alliance (LSA) as a appropriate venue for your research. We look forward to receiving your revised manuscript.

Sincerely,

Shachi Bhatt, Ph.D.
Executive Editor
Life Science Alliance

A. THESE ITEMS ARE REQUIRED FOR REVISIONS

-- A letter addressing the reviewers' comments point by point.

-- An editable version of the final text (.DOC or .DOCX) is needed for copyediting (no PDFs).

-- High-resolution figure, supplementary figure and video files uploaded as individual files: See our detailed guidelines for preparing your production-ready images, https://www.life-science-alliance.org/authors

-- Summary blurb (enter in submission system): A short text summarizing in a single sentence the study (max. 200 characters including spaces). This text is used in conjunction with the titles of papers, hence should be informative and complementary to the title and running title. It should describe the context and significance of the findings for a general readership; it should be written in the present tense and refer to the work in the third person. Author names should not be mentioned.

B. MANUSCRIPT ORGANIZATION AND FORMATTING:

Full guidelines are available on our Instructions for Authors page, https://www.life-science-alliance.org/authors

We encourage our authors to provide original source data, particularly uncropped/-processed electrophoretic blots and spreadsheets for the main figures of the manuscript. If you would like to add source data, we would welcome one PDF/Excel-file per figure for this information. These files will be linked online as supplementary "Source Data" files.

IMPORTANT: It is Life Science Alliance policy that if requested, original data images must be made available. Failure to provide original images upon request will result in unavoidable delays in publication. Please ensure that you have access to all original microscopy and blot data images before submitting your revision.

RE: Life Science Alliance Manuscript #LSA-2020-00918-TR

Dr. Adam L Hughes
University of Utah
Biochemistry
15 N Medical Drive East
RM 4100
Salt Lake City, Utah 84112

Dear Dr. Hughes,

Thank you for submitting your revised manuscript entitled "ER-targeting of non-imported mitochondrial proteins is dependent on the GET pathway". We would be happy to publish your paper in Life Science Alliance pending final textual revisions as requested by our 2 arbitrator reviewers, and additional edits necessary to meet our formatting guidelines.

Along with the points listed below, please also attend to the following,

-please upload your main and supplementary figures as single files

-please add your Table legends to the main manuscript text

-please add a callout for Figure 4D and Table S2 in your main manuscript text

-please use the [10 author names, et al.] format in your references (i.e. limit the author names to the first 10)

If you are planning a press release on your work, please inform us immediately to allow informing our production team and scheduling a release date.

To upload the final version of your manuscript, please log in to your account: https://lsa.msubmit.net/cgi-bin/main.plex

You will be guided to complete the submission of your revised manuscript and to fill in all necessary information. Please get in touch in case you do not know or remember your login name.

To avoid unnecessary delays in the acceptance and publication of your paper, please read the following information carefully.

A. FINAL FILES:

These items are required for acceptance.

-- An editable version of the final text (.DOC or .DOCX) is needed for copyediting (no PDFs).

-- High-resolution figure, supplementary figure and video files uploaded as individual files: See our detailed guidelines for preparing your production-ready images, https://www.life-science-

alliance.org/authors

-- Summary blurb (enter in submission system): A short text summarizing in a single sentence the study (max. 200 characters including spaces). This text is used in conjunction with the titles of papers, hence should be informative and complementary to the title. It should describe the context and significance of the findings for a general readership; it should be written in the present tense and refer to the work in the third person. Author names should not be mentioned.

B. MANUSCRIPT ORGANIZATION AND FORMATTING:

Full guidelines are available on our Instructions for Authors page, https://www.life-science-alliance.org/authors

We encourage our authors to provide original source data, particularly uncropped/-processed electrophoretic blots and spreadsheets for the main figures of the manuscript. If you would like to add source data, we would welcome one PDF/Excel-file per figure for this information. These files will be linked online as supplementary "Source Data" files.

Submission of a paper that does not conform to Life Science Alliance guidelines will delay the acceptance of your manuscript.

It is Life Science Alliance policy that if requested, original data images must be made available to the editors. Failure to provide original images upon request will result in unavoidable delays in publication. Please ensure that you have access to all original data images prior to final submission.

The license to publish form must be signed before your manuscript can be sent to production. A link to the electronic license to publish form will be sent to the corresponding author only. Please take a moment to check your funder requirements.

Reviews, decision letters, and point-by-point responses associated with peer-review at Life Science Alliance will be published online, alongside the manuscript. If you do want to opt out of having the reviewer reports and your point-by-point responses displayed, please let us know immediately.

Thank you for your attention to these final processing requirements. Please revise and format the manuscript and upload materials within 7 days.

Thank you for this interesting contribution, we look forward to publishing your paper in Life Science Alliance.

Sincerely,
Shachi Bhatt, Ph.D.
Executive Editor
Life Science Alliance
https://www.lsajournal.org/ Tweet @SciBhatt @LSAjournal

January 6, 2021

RE: Life Science Alliance Manuscript #LSA-2020-00918-TRR

Dr. Adam L Hughes
University of Utah
Biochemistry
15 N Medical Drive East
RM 4100
Salt Lake City, Utah 84112

Dear Dr. Hughes,

Thank you for submitting your Research Article entitled "ER-targeting of non-imported mitochondrial carrier proteins is dependent on the GET pathway". It is a pleasure to let you know that your manuscript is now accepted for publication in Life Science Alliance. Congratulations on this interesting work.

The final published version of your manuscript will be deposited by us to PubMed Central upon online publication.

Your manuscript will now progress through copyediting and proofing. It is journal policy that authors provide original data upon request.

Reviews, decision letters, and point-by-point responses associated with peer-review at Life Science Alliance will be published online, alongside the manuscript. If you do want to opt out of having the reviewer reports and your point-by-point responses displayed, please let us know immediately.

IMPORTANT: If you will be unreachable at any time, please provide us with the email address of an alternate author. Failure to respond to routine queries may lead to unavoidable delays in publication.

Scheduling details will be available from our production department. You will receive proofs shortly before the publication date. Only essential corrections can be made at the proof stage so if there are any minor final changes you wish to make to the manuscript, please let the journal office know now.

DISTRIBUTION OF MATERIALS:

Authors are required to distribute freely any materials used in experiments published in Life Science Alliance. Authors are encouraged to deposit materials used in their studies to the appropriate repositories for distribution to researchers.

You can contact the journal office with any questions, contact@life-science-alliance.org

Again, congratulations on a very nice paper. I hope you found the review process to be constructive and are pleased with how the manuscript was handled editorially. We look forward to future exciting submissions from your lab.

Sincerely,

Shachi Bhatt, Ph.D.
Executive Editor
Life Science Alliance
https://www.lsajournal.org/
Tweet @SciBhatt @LSAjournal
